# Physiological Responses During High-Intensity Interval Training in Young Swimmers

**DOI:** 10.3389/fphys.2021.662029

**Published:** 2021-07-01

**Authors:** Tiago André Freire Almeida, Dalton Müller Pessôa Filho, Mário Cunha Espada, Joana Filipa Reis, Andrei Sancassani, Danilo Alexandre Massini, Fernando Jorge Santos, Francisco Besone Alves

**Affiliations:** ^1^Interdisciplinary Center for the Study of Human Performance (CIPER), Faculdade de Motricidade Humana, Universidade de Lisboa, Lisbon, Portugal; ^2^Faculdade de Motricidade Humana, Universidade de Lisboa, Lisbon, Portugal; ^3^Department of Physical Education, São Paulo State University (UNESP), Bauru, Brazil; ^4^Institute of Bioscience, Graduate Program in Human Development and Technology, São Paulo State University (UNESP), Rio Claro, Brazil; ^5^Department of Science and Technology, Polytechnic Institute of Setúbal, Setúbal, Portugal; ^6^Quality of Life Research Center, Santarém, Portugal

**Keywords:** interval training, oxygen uptake kinetics, work-interval, performance, swimming

## Abstract

This study analyzed whether 100- and 200-m interval training (IT) in swimming differed regarding temporal, perceptual, and physiological responses. The IT was performed at maximal aerobic velocity (MAV) until exhaustion and time spent near to maximalVO_2_ peak oxygen uptake (⩒O_2_peak), total time limit (t_Lim_), peak blood lactate [La^−^] peak, ⩒O_2_ kinetics (⩒O_2_K), and rate of perceived exertion (RPE) were compared between protocols. Twelve swimmers (seven males 16.1 ± 1.1 and five females 14.2 ± 1 years) completed a discontinuous incremental step test for the second ventilatory threshold (VT_2_), ⩒O_2_peak, and MAV assessment. The swimmers subsequently completed two IT protocols at MAV with 100- and 200-m bouts to determine the maximal ⩒O_2_ (peak-⩒O_2_) and time spent ≥VT_2_, 90, and 95% of ⩒O_2_peak for the entire protocols (IT_100_ and IT_200_) and during the first 800-m of each protocol (IT_8x100_ and IT_4x200_). A portable apparatus (K4b^2^) sampled gas exchange through a snorkel and an underwater led signal controlled the velocity. RPE was also recorded. The Peak-⩒O_2_ attained during IT_8x100_ and IT_4x200_ (57.3 ± 4.9 vs. 57.2 ± 4.6 ml·kg^−1^·min^−1^) were not different between protocols (*p* = 0.98) nor to ⩒O_2_peak (59.2 ± 4.2 ml·kg^−1^·min^−1^, *p* = 0.37). The time constant of ⩒O_2_K (24.9 ± 8.4 vs. 25.1 ± 6.3-s, *p* = 0.67) and [La^−^] peak (7.9 ± 3.4 and 8.7 ± 1.5 mmol·L^−1^, *p* = 0.15) also did not differ between IT_100_ and IT_200_. The time spent ≥VT_2_, 90, and 95%⩒O_2_peak were also not different between IT_8x100_ and IT_4x200_ (*p* = 0.93, 0.63, and 1.00, respectively). The RPE for IT_8x100_ was lower than that for IT_4x200_ (7.62 ± 2 vs. 9.5 ± 0.7, *p* = 0.01). Both protocols are considered suitable for aerobic power enhancement, since ⩒O_2_peak was attained with similar ⩒O_2_K and sustained with no differences in t_Lim_. However, the fact that only the RPE differed between the IT protocols suggested that coaches should consider that nx100-m/15-s is perceived as less difficult to perform compared with nx200-m/30-s for the first 800-m when managing the best strategy to be implemented for aerobic power training.

## Introduction

Interval training (IT) has been considered an effective exercise plan to improve endurance performance and maximal aerobic velocity (MAV, i.e., the velocity corresponding to the peak oxygen uptake, ⩒O_2_peak; Billat and Koralsztein, 1996; Billat et al., 2000; Dalamitros et al., 2016), and, therefore, has been proposed as a successful way to enhance cardiovascular and muscle adjustments needed to optimize performance during middle-distance racing in different sports, e.g., running and swimming (Billat, 2001; Libicz et al., 2005; Reis et al., 2012a,b; Espada et al., 2015, 2021). The time sustained with ⩒O_2_ responses closer to the maximal rates (90–100% of ⩒O_2_peak) is considered an important factor to maximize aerobic training benefits (⩒O_2_peak, O_2_ transport, and mitochondrial density) and avoid high oxygen deficits and fast metabolite accumulation, which can contribute to an increase in endurance capacity and tolerance at severe and maximal intensities (Billat and Koralsztein, 1996; Millet et al., 2003a,b; Bentley et al., 2005; Sousa et al., 2017).

The IT planning requires the organization of several parameters, such as work intensity, distance and duration, rest mode (active or passive) and duration, number of bouts to be performed (*n* repetitions), number of sets, and the duration of recovery between sets (Buchheit and Laursen, 2013a,b). When IT is planned to increase the time limit at MAV or/and to ensure an increase in time exercising closer to ⩒O_2_peak response, workouts have been designed with repeated bouts lasting 2–4 min, which is characterized as long-term work intervals (Buchheit and Laursen, 2013b; Wen et al., 2019). However, performing short-duration work intervals (<60 s) could allow the athlete to complete longer IT sessions with greater oxidative demands and lower anaerobic glycolytic contribution than long work intervals, despite the similarities between short and long work intervals regarding the time spent at ⩒O_2_peak (Zuniga et al., 2011; Rønnestad and Hansen, 2016) and the effectiveness for improving ⩒O_2_peak (Wen et al., 2019). Hence, the planning of work interval duration must consider the energetic balance that matches the specificity of the race to be performed.

When performing continuous exercise at MAV, the time limit approximates to ~5 min for different exercise modes (running, cycling, swimming, and paddling; Billat et al., 1996a). However, IT has been reported to increase significantly the time limit and time spent at high ⩒O_2_ when designed either with short- or long-distance work intervals at 1:1 ratio (30:30 or 120:120 s) but with higher blood lactate accumulation (>3 mmol·L^−1^) and oxygen deficit (>~5 ml·kg^−1^) when using the latter (Billat, 2001; Zuniga et al., 2011; Buchheit and Laursen, 2013b). In swimming, short-distance work intervals (*n* × 100-m) performed at submaximal or maximal velocities (≤95 or 100% MAV) have been shown to induce higher (absolute) time limit and time spent at submaximal or maximal ⩒O_2_ (>90 or 100% ⩒O_2_peak) than a single trial performed at same velocities (Bentley et al., 2005; Libicz et al., 2005; Sousa et al., 2017). Although the literature is not extensive, the temporal and ⩒O_2_ responses during IT in swimming, seems to point out that using 60–120-s work intervals at velocities ≥95% of MAV is recommended to stimulate improvements in aerobic power and endurance in high swimming intensity (Dalamitros et al., 2016; Sousa et al., 2017).

However, there are still doubts on how to define the IT to provide the best combination of aerobic and anaerobic responses in swimming, especially considering the requirements for successful performance in middle-distance events, as proposed for running and cycling (Billat, 2001; Spencer and Gastin, 2001; Buchheit and Laursen, 2013b). In swimming, performing IT at MAV with 1:1 or 1:1/2 ratios for work:rest elicits only moderate blood lactate accumulation, clearly lower values than those reported for running and cycling (Billat et al., 2000; Zuniga et al., 2011), which is probably attributed to the clearance mechanism during long rest periods (Bentley et al., 2005; Libicz et al., 2005). Therefore, we could expect that longer work intervals or decreases in the rest periods would lead to higher anaerobic glycolic energy release (Buchheit and Laursen, 2013b). However, this has not been studied in swimming.

⩒O_2_ kinetics (⩒O_2_K) has been associated with endurance performance (Jones and Burnley, 2009; Reis et al., 2012b; Espada et al., 2015; Almeida et al., 2020) and time spent at ⩒O_2_max (Millet et al., 2003a,b; Sousa et al., 2018), since faster kinetics can represent an accelerated oxidative rate. It has been reported that athletes with faster ⩒O_2_K can reach ⩒O_2_peak faster and present lower oxygen deficits (Millet et al., 2003b). However, Bentley et al. (2005) did not find any influence of faster kinetics with the time spent near ⩒O_2_ maximal values on swimmers when performing IT with 400-m bouts. Furthermore, Sousa et al. (2015) reported that swimmers seem to have slower ⩒O_2_K than runners and cyclists, which can indicate that IT in swimming could require longer work intervals to induce near maximal ⩒O_2_ responses.

Considering that different combinations of the IT parameters truly induce different acute physiological responses (time spent closer to maximal ⩒O_2_), it is crucial to investigate different types of IT. Therefore, to understand whether different combinations of IT produce different but high aerobic and anaerobic responses, while exercising at MAV, this study compared the ⩒O_2_, blood lactate accumulation, oxygen deficit, and rate of perceived exertion (RPE) responses during two different ITs, designed with 100- (IT_100_) and 200-m (IT_200_) swimming bouts, until exhaustion. The first 800 m of each IT session was also considered for analysis in order to allow a direct comparison between training sets (IT_8x100_ vs. IT_4x200_). We chose this format for IT in an attempt to represent the usual intermittent bouts in the daily training routine and therefore expected an analysis that is more ecological.

We hypothesize that both ITs will elicit the achievement of ⩒O_2peak_; however, IT_100_ will present longer times to exhaustion and consequently longer times spent near swimmers ⩒O_2_ maximal values, and swimmers with faster ⩒O_2_K will also present longer times to exhaustion and times spent near ⩒O_2_peak.

## Materials and Methods

### Experimental Design

To analyze the physiological and temporal responses during two different intermittent swimming (IT) protocols, the peak oxygen uptake (⩒O_2_peak), second ventilatory threshold (VT₂), and MAV were assessed by a discontinuous incremental step test performed until a maximal 200-m pace or to volitional exhaustion. In a randomized order, the swimmers performed two different IT protocols until exhaustion at MAV, consisting of 100 or 200-m repetitions, to compare the ventilatory and physiological responses between the two IT formats. All the swimmers performed the three testing protocols in front crawl swimming with in-water starts and open turns without underwater gliding (in a 25-m swimming pool), with gas exchange analysis recorded by a portable gas analyzer (K4b^2^, Cosmed®, Rome, Italy) connected to the swimmers by a respiratory snorkel and a valve system (new-AquaTrainer®, Cosmed, Rome, Italy). The transportation of this system along the swimming pool can be watching in the Supplementary Material.

The participants were instructed to report to the swimming pool well hydrated, fed, and to abstain from caffeine, alcohol, and strenuous exercise in the 24 h preceding the testing protocols. The same environmental conditions (time of day ± 2 h, water temperature ~28°C, and relative humidity ~50%) and same pre-test warm up protocol were ensured for all tests in order to minimize the effects of circadian rhythms and differences in prior exercise. The sessions were performed in the beginning of the preparatory period of the second macrocycle of competitive season of the swimmers, after a period of 2 weeks for training adaptation and were separated by at least 48 h.

### Participants

Twelve well-trained young swimmers (seven males and five females) were informed about the procedures and experimental risks of the study and signed a written informed consent (and their legal guardians when under 18 years old). All the swimmers were fully familiarized with the equipment and procedures before the beginning of the tests. The recruited swimmers had to be regularly competing in state or national championships for a minimum of 3 years prior to the beginning of the study, as a criterion to participate in this study. This study was approved by the local University Ethical Committee (CEFMH: 39/2015) and was conducted in accordance with the 1964 Declaration of Helsinki (Harriss et al., 2017). The descriptive characteristics of the swimmers are presented in Table 1.

**Table 1:** Anthropometric (mean ± SD) characteristics of the participants.

Variables	Male (*N* = 7)	Female (*N* = 5)	Group (*N* = 12)
Age (yrs)	16.1 ± 1.1	14.2 ± 1.0	15.3 ± 1.4
Height (m)	1.76 ± 0.1	1.58 ± 0.1	1.69 ± 0.1
Total body mass (kg)	64.8 ± 7.8	50.6 ± 5.1	58.9 ± 9.8

### Incremental Step Test and Interval Training Protocol

The discontinuous incremental step test was structured with 6 × 250 and 1 × 200-m steps performed with 30-s rest for blood lactate sampling (Espada et al., 2015; Almeida et al., 2020). The velocity started at 50% of the velocity at 200-m (v200-m) maximal performance, which was performed 48 h before the execution of the incremental step test and ensured the similar swimming mode (in water starting, open turns and no underwater gliding). The following steps were incremented at 55, 60, 70, 80, 90, and 100% rates of v200-m, aiming to ensure a narrow rest-to-work transition for the three initial steps and therefore enabling ideal warming with no metabolism compromise (premature acidosis and glycogen depletion) for the remaining steps (Espada et al., 2015; Almeida et al., 2020).

In a randomized order, the swimmers performed two different IT swimming protocols at MAV 48 h after the incremental test and 48 h apart from each other. The IT was performed until voluntary exhaustion, following the protocols: (1) *n* repetitions of 100-m interspersed by 15-s of rest (IT_100_), and (2) *n* repetitions of 200-m interspersed by 30-s of rest (IT_200_). The comparison between each IT protocol considered the first 800 m (IT_8x100_ and IT_4x200_, respectively for the IT_100_ and IT_200_), as well as the entire IT_100_ and IT_200_ protocols, analyzing temporal, perceptual, and physiological responses.

**Figure 1:**
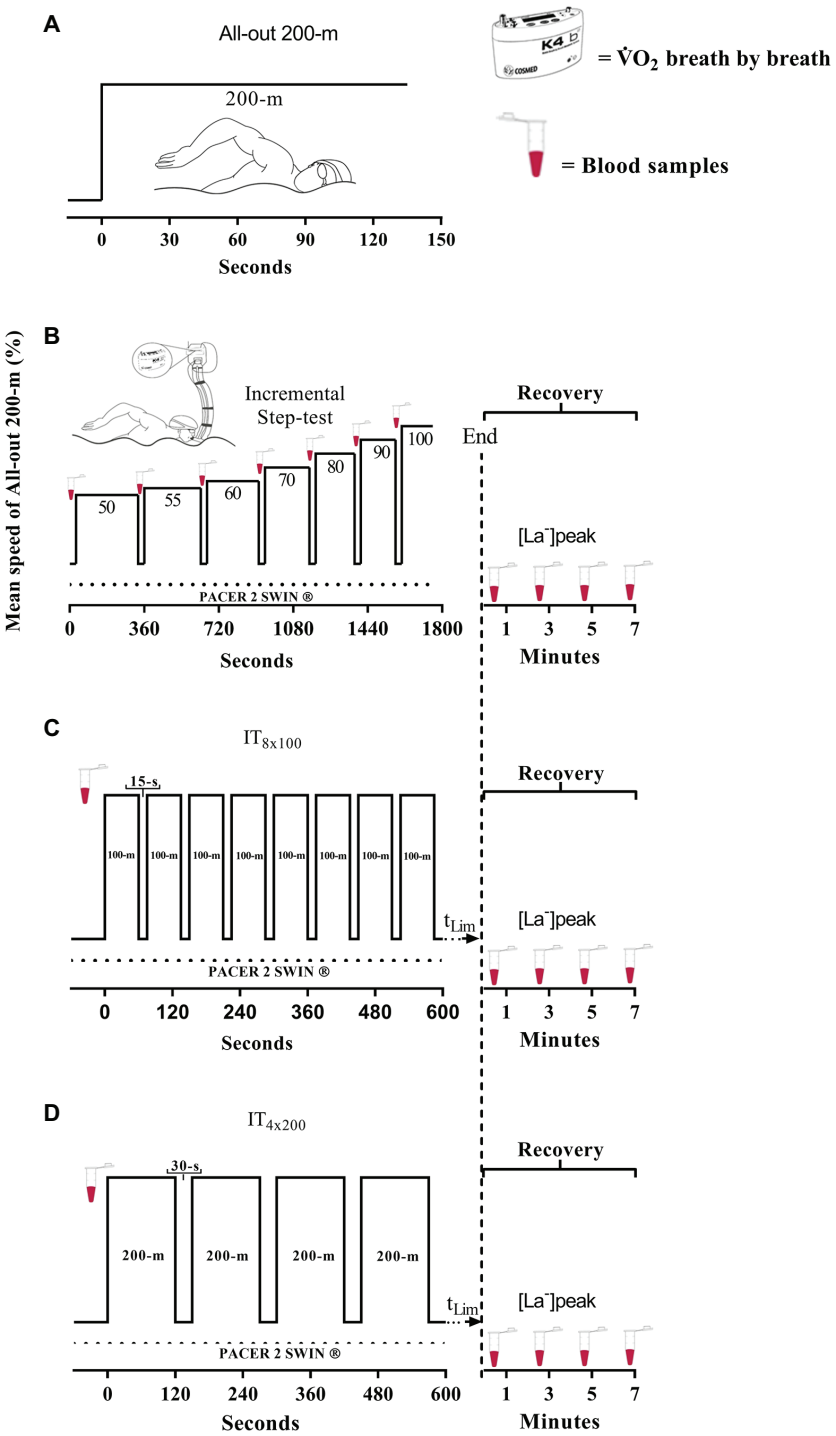
Overview of experimental design for 200-m performance **(A)**, discontinuous incremental step test **(B)**, IT_8x100_ **(C)**, and IT_4x200_ **(D)**. The total time limit (t_Lim_) indicates *n* repetitions until exhaustion during IT100 and IT200, respectively (**A**,**B**).

For the control of swimming velocity during each step of the incremental test and during each *n* repetition of IT_100_ and IT_200_, an underwater visual pacer was employed, which was designed with 26 led lights that subsequently signaled the pacing (Pacer2Swim®, KulzerTEC, Santa Maria da Feira, Portugal), and was used to provide the swimmers an accurate notion of the correct velocity for each test. Figure 1 depicts an overall view of the IT protocols.

### Measurements and ⩒O_2_ Kinetics

For the gas exchange analysis, a telemetric portable gas analyzer (K4b^2^, Cosmed®, Rome, Italy) was connected to the swimmers by a respiratory snorkel and a valve system (new-AquaTrainer®, Cosmed, Rome, Italy), allowing breath-by-breath pulmonary gas collection (Reis et al., 2010; Baldari et al., 2013). The system was moved alongside the swimmers by a member of the research team. Before the start (10 min of resting), during, and after each protocol (at 1, 3, 5, and 7-min in the recovery phase) capillary blood samples (25 μl) were collected from the earlobe (carefully dried before each sampling) for blood lactate [La^−^] analysis (YSI, 2300 STAT®, Yellow Springs, Ohio). Exceptionally during the IT protocols, the blood samples were collected before (at rest) and after (at recovery) only. The peak of [La^−^] concentration ([La^−^]peak) was measured in the recovery phase after the incremental step test and each IT protocol. The RPE was recorded through the CR-10 scale of Borg (1990).

During the incremental step test, the ⩒O_2_peak was measured as the highest 30-s (moving) averaged ⩒O_2_ in each step, and MAV was considered as the velocity corresponding to the step where ⩒O_2_peak occurred (Billat and Koralsztein, 1996). VT₂ was determined by gas analysis in the incremental test according to the recommendations of Filho et al. (2012), and was examined visually using the responses from the V̇_E_/V̇CO_2_, V̇_E_/⩒O_2_, P_ET_CO_2_, and P_ET_O_2_ parameters. The criterion was the continuous increase in V̇_E_/⩒O_2_ and V̇_E_/V̇CO_2_ ratio curves related to the reduction in P_ET_CO_2_. The point of VT_2_ localization was observed by two independent experts. Swimming velocity at VT_2_ corresponded to the incremental testing step at which VT_2_ occurred. Maximal exertion during the incremental step test was ensured by analyzing secondary criteria, as [La^−^]peak (≥8 mmol·l^−1^) and respiratory exchange ratio (RER > 1; Baldari et al., 2013). The maximal 30-s (moving) averaged ⩒O_2_ attained during each IT protocol was considered the Peak-⩒O_2_, and the MPeak-⩒O_2_ was the average of the maximal ⩒O_2_ (30-s moving average) attained during each bout of the IT protocols. Both Peak-⩒O_2_ and MPeak-⩒O_2_ were calculated in IT_8x100_ and IT_4x200_, as well as for the entire IT_100_ and IT_200_.

The time spent (in seconds) with ⩒O_2_ above the VT₂(t@VT_2_), 90% (t@90%), and 95% (t@95%) of ⩒O_2peak_ and the corresponding percentage (%) for the total duration of each IT were determined, as well as the time to exhaustion (t_Lim_) and distance performed by each swimmer.

For the ⩒O_2_K analysis, the outliers (exclusion of values lying over three SDs from the local mean) were previously excluded from the analysis, and the breath-by-breath data were interpolated into 1-s values. Only the first bout of each IT protocol (100 and 200-m) was used for the determination of the ⩒O_2_K parameters [time delay (TD), time constant (τ), and amplitude (A)]. The cardiodynamic phase of the ⩒O_2_ response at the onset of the exercise was discharged by removing the first 20 s of the ⩒O_2_ response (Filho et al., 2012). As described by Reis et al. (2010), an individual “snorkel delay” (ISD) that corresponded to the difference between the onset of exercise and the time when the following breaths summed up a tidal volume superior to the outlet tube volume was calculated for each test. The ISD was adapted to the specific characteristic of the snorkel device used in this study and then integrated into the time delay of the ⩒O_2_ response. The ⩒O_2_ vs. time mono-exponential adjustments were analyzed through an iterative procedure by minimizing the sum of the mean squares of the differences between the modeled and the measured ⩒O_2_ values, according to the following equation:

$$⩒O_{2\left(t\right)}=⩒O_{\mathrm{2base}}+A•\left(1-e^{-\left(t-\mathrm{TD}\right)/\tau }\right)$$

where ⩒O_2(t)_ represents the relative ⩒O_2_ at a given time; ⩒O_2_base represents the ⩒O_2_ at rest, which was calculated as the average of the first 30-s of the last minute before the start of the exercise (after 10-min of passive rest); TD, τ, and A represent the time delay, time constant, and amplitude of the primary phase of the ⩒O_2_response, respectively (Rodríguez et al., 2003; Sousa et al., 2013; Almeida et al., 2020). The oxygen deficit (O_2Def_) at the onset of the first 100 and 200-m of each IT protocol was measured as the product between mean response time (MRT) and A, where the MRT is TD × τ (Whipp et al., 2005).

### Statistical Analysis

Initially, normality and homogeneity of data were accessed by the Shapiro–Wilk and Levene tests. The comparison of the temporal, perceptual, and physiological responses between the two IT protocols was performed considering all the samples with the *t*-test for unpaired samples, or with the Mann–Whitney test when the assumptions for parametric tests were not met. The Kruskal–Wallis test compared ⩒O_2_peak and [La^−^]peak responses during the incremental step test vs. Peak-⩒O_2_ during IT_8x100_ and IT_4x200_, and vs. Peak-⩒O_2_ during the entire IT_100_ and IT_200_ protocols. The Spearman coefficient (*ro*) tested the rank-order correlation between physiological, perceptual, and temporal responses during the IT protocols. The effect sizes (ES) were calculated by Cliff’s δ, considering the *n* and *p* values for the differences analyzed by the Mann–Whitney test, which was interpreted as 0.2 weak, 0.36 medium, 0.52 strong, and 0.76 very strong (Sheskin, 2011). The *ro* was interpreted as <0.2 (trivial), 0.2–0.49 (small), 0.5–0.8 (medium), and >0.8 (large; Ferguson, 2009). All statistical analyses were performed with the Statistical Package for the Social Sciences (version 25.0; SPSS®, Chicago, IL, United States), and statistical significance was accepted at *p* ≤ 0.05.

## Results

**Figure 2:**
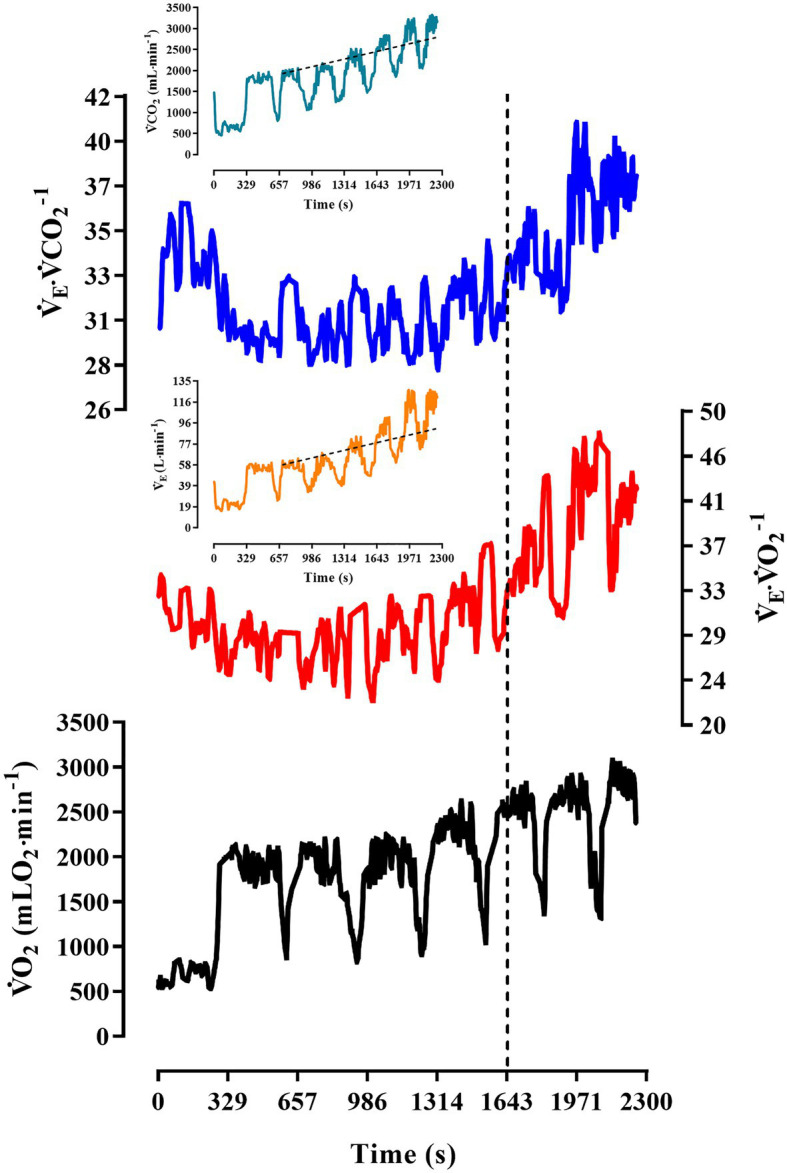
Pulmonary ⩒O_2_ and other gas-exchange responses during the incremental step test for a male participant. The vertical dashed line indicates VT_2_ occurrence and the inclined dashed line illustrates isocapnic disturbance and ventilatory compensation. The progression for this male swimmer ranged from 67 to 100%⩒O_2_peak, and time performance range was 265–161-s, respectively, from the first to the seventh step.

The physiological responses of the swimmers during the incremental step test are shown in Table 2. The [La^−^]peak and RER reached values corresponding to the maximal exertion, and the entire sample of participants (male and female) exhibited no large variance for maximal and submaximal indexes of aerobic conditioning level [coefficient of variation (CV) < 10% for ⩒O_2_peak and VT_2_]. An individual response of ⩒O_2_ increasing during the incremental step test and the profile of gas-exchange variable (⩒_E_, ⩒CO_2_, ⩒_E_/VO_2_, and ⩒_E_/⩒CO_2_) matching VT_2_ criteria are illustrated in Figure 2.

**Table 2:** Measurements (mean ± SD) during the incremental step test for the entire groups of participants (*N* = 12).

Variables	Mean ± SD	IC95%	SEM
⩒O_2_peak (ml·kg^−1^·min^−1^)	59.2 ± 4.2	56.5–61.8	1.20
MAV (m·s^−1^)	1.27 ± 0.09	1.21–1.32	0.03
VT_2_ (ml·kg^−1^·min^−1^)	52.0 ± 3.9	49.5–54.4	1.14
VT_2_ (%⩒O_2_peak)	87.9 ± 3.2	85.8–89.9	0.93
vVT_2_ (m·s^−1^)	1.20 ± 0.10	1.14–1.26	0.03
vVT_2_ (%MAV)	94.0 ± 3.9	91.5–96.4	1.11
[La-]peak (mmol·L^−1^)	10.3 ± 2.6	8.6–11.9	0.74
RER	1.05 ± 0.15	0.96–1.15	0.04

IC95%, confidence interval; SEM, standard error of mean; MAV, maximal aerobic power; and RER, respiratory exchange ratio.

**Figure 3:**
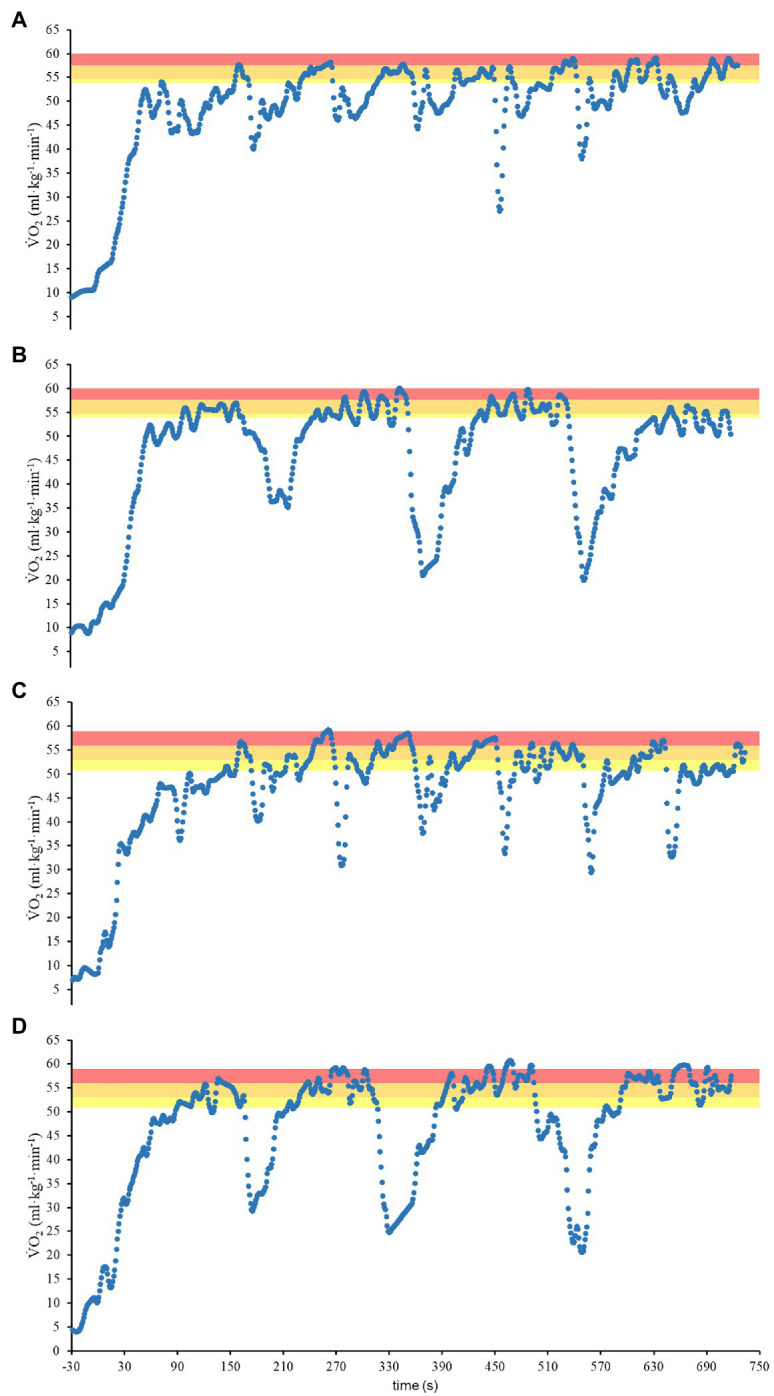
⩒O_2_ response during IT_8x100_ **(A,C)** and IT_4x200_ **(B,D)** for male **(A,B)** and female **(C,D)** swimmers. The yellow, orange, and red shadow areas highlight the t@VT_2_, t@90%, and t@95% of ⩒O_2peak_, respectively.

The physiological and perceptual responses during the IT protocols and ⩒O_2_K analysis are shown in Table 3. Typical responses of ⩒O_2_ in IT_8x100_ and IT_4x200_ are illustrated in Figure 3 for the male (panels A and B) and female (panels C and D) swimmers. The velocities while performing IT_100_ and IT_200_ did not differ from MAV (*p* = 0.89 and *p* = 0.39, respectively) or between each other (*p* = 0.44). When comparing ⩒O_2_peak vs. Peak-⩒O_2_ (IT_8x100_) vs. Peak-⩒O_2_ (IT_4x200_), no significant differences were observed (*p* = 0.37). However, differences were observed for the comparison of ⩒O_2_peak vs. MPeak-⩒O_2_ (IT_8x100_) vs. MPeak-⩒O_2_ (IT_4x200_; *p* = 0.01). Similar results were observed when comparing the IT_100_ and IT_200_ protocols. Therefore, there were no significant differences for ⩒O_2_peak vs. Peak-⩒O_2_ (IT_100_) vs. Peak-⩒O_2_ (IT_200_; *p* = 0.32), but significant differences were observed for ⩒O_2_peak vs. MPeak-⩒O_2_ (IT_100_) vs. MPeak-⩒O_2_ (IT_200_; *p* < 0.01). Additionally, no significant differences were observed for [La^−^]peak responses after the incremental step test vs. IT_100_ vs. IT_200_ (*p* = 0.15). Regarding the RPE, differences were observed when comparing IT_8x100_ vs. IT_4x200_ (*p* = 0.012; δ = 0.36) but no differences for IT100 vs. IT200 (*p* = 0.55). The measurements of A_1_ (*p* = 0.38), TD (*p* = 0.89), τ (*p* = 0.67), and O_2Def_ (*p* = 0.98) did not differ between IT_100_ and IT_200_.

**Table 3:** Mean ± SD of the physiological and perceptual responses during the IT protocols, and ⩒O2 kinetics (⩒O_2_K) during the first bout of IT_100_ and IT_200_ (*N* = 12).

Variable	IT_8x100_	IT_4x200_	IT_100_	IT_200_
Peak-⩒O_2_ (ml·kg^−1^·min^−1^)	57.3 ± 4.9	57.2 ± 4.6	57.5 ± 5.0	57.3 ± 4.4
Peak-⩒O_2_ (%⩒O_2_peak)	96.8 ± 5.8	96.7 ± 4.4	97.1 ± 5.9	96.8 ± 3.8
MPeak-⩒O_2_ (ml·kg^−1^·min^−1^)	54.5 ± 4.2	55.2 ± 4.0	54.3 ± 4.1	55.1 ± 4.1
MPeak-⩒O_2_ (%⩒O_2_peak)	92.1 ± 4.6	93.3 ± 4.5	91.8 ± 4.6	93.3 ± 4.8
[La^−^]peak (mmol·L^−1^)	-	-	7.9 ± 3.4	8.7 ± 1.5
RPE (0–10 units)	7.62 ± 2.0^*^	9.5 ± 0.7^*^	9.4 ± 0.9	9.7 ± 0.9
A (ml·kg^−1^·min^−1^)	-	-	44.9 ± 6.1	43.0 ± 4.6
TD (s)			11.7 ± 4.2	11.1 ± 3.0
τ(s)	-	-	24.2 ± 8.9	24.6 ± 6.6
O_2Def_ (ml·kg^−1^·min^−1^)	-	-	28.8 ± 14.3	27.0 ± 8.5
Velocity (m/s)	-	-	1.26 ± 0.09	1.23 ± 0.09
Velocity (%MAV)	-	-	99.7 ± 2.6	96.8 ± 2.4

Peak-⩒O_2_ and %Peak-⩒O_2_: maximal ⩒O_2_ achieved in the test and corresponding percentage of ⩒O_2_peak; MPeak-⩒O_2_ and %MPeak-⩒O_2_: average value and corresponding percentage of the Peak-⩒O_2_ achieved during the performance of each bout of IT protocols; [La^−^]peak: peak blood lactate concentration after each IT protocol performance; velocity: the actual velocity while performing each IT protocol.

* Statistical difference between IT protocols (IT_8x100_ vs. IT_4x200_ or IT_100_ vs. IT_200_) at *p* ≤ 0.05.

**Figure 4:**
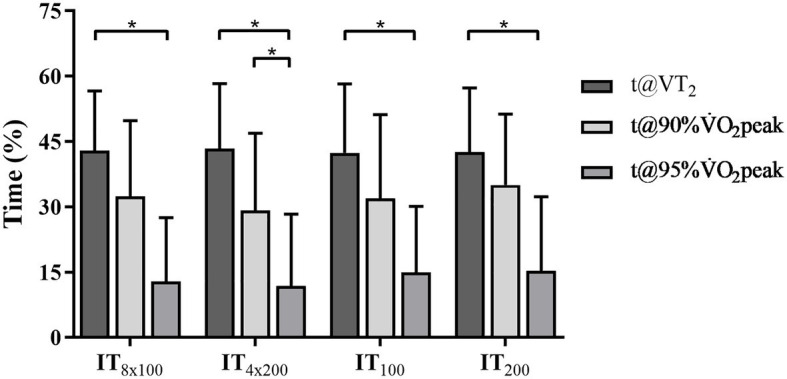
The percentage of t@VT_2_, t@90%⩒O_2_peak, and t@95%⩒O_2_peak at IT_8x100_, IT_4x200_, IT_100_, and IT_200_. ^*^Statistical difference intra interval training (IT) protocols (t@VT_2_ vs. t@90%⩒O_2_peak vs. t@95%⩒O_2_peak) at *p* ≤ 0.05. No differences were observed between the IT protocols (IT_8x100_ vs. IT_4x200_ or IT_100_ vs. IT_200_).

The values of distance, time limit, and time spent above VT₂, 90, and 95% of ⩒O_2_peak during the IT protocols are shown in Table 4. The comparison between the percentage of t@VT₂, t@90%, and t@95% of ⩒O_2_peak during each IT protocol are illustrated in Figure 4. There were no significant differences in distance (*p* = 0.09) and t_Lim_ (*p* = 0.16) between IT_100_ and IT_200_. No significant differences were observed when comparing IT_8x100_ vs. IT_4x200_ with regard to t@VT₂ (*p* = 0.72), t@90% (*p* = 0.63), and t@95% (*p* = 1). Similarly, IT_100_ vs. IT_200_ did not differ regarding t@VT₂ (*p* = 0.22), t@90% (*p* = 0.29), and t@95% (*p* = 0.16). However, t@VT_2_ was higher than t@95% during IT_8x100_ (*p* < 0.01) and IT_4x200_ (*p* < 0.01).

**Table 4:** Mean ± SD of the distance and time performance during the IT protocols (*N* = 12).

Variable	IT_8x100_	IT_4x200_	IT_100_	IT_200_
Distance (m)	800	800	1308.3 ± 611.7	1016.7 ± 403.8
t_Lim_ (s)	-	-	1034.8 ± 462.8	826.1 ± 302.7
t@VT_2_ (s)	274.7 ± 89.9^†^	290.1 ± 104.9^†^	412.8 ± 202.6	325.2 ± 109.5
t@90%⩒O_2_peak (s)	208.0 ± 123.5	218.4 ± 122.1	306.9 ± 216.4	234.4 ± 119.9
t@95%⩒O_2_peak (s)	97.3 ± 100.1^†^	86.2 ± 109.1^†^	147.5 ± 143.1	103.8 ± 120.5

t@VT_2_, t@90%⩒O_2_peak, and t@95%⩒O_2_peak: time spent with the rate of ⩒O_2_ response at or above VT_2_, 90, and 95% of ⩒O_2_peak.

† Statistical difference between IT protocols (IT_8x100_ vs. IT_4x200_ or IT_100_ vs. IT_200_) at *p* ≤ 0.05.

The correlation between the IT protocols with regard to the temporal, physiological, and perceptual responses is shown in Table 5. The total distance and t_Lim_ attained during IT_100_ and IT_200_ did not associate the protocols with each other (*p* = 0.11 and *p* = 0.27) regarding performance. However, the [La^−^]peaks after IT_100_ and IT_200_ are positively correlated (*p* < 0.01), as well as τ and O_2Def_ for ⩒O_2_K during the first bout of IT_100_ and IT_200_ (*p* = 0.03 and *p* = 0.05), despite Peak-⩒O_2_ not being associated when analyzing IT_100_ vs. IT_200_ (*p* = 0.53) and IT_8x100_ vs. IT_4x200_ (*p* = 0.6). Therefore, the IT protocols are associated with each other only with regard to some features of anaerobic contribution and initial ⩒O_2_ response. The t_Lim_ at IT_100_ and IT_200_ correlated with RPE at the strong (*p* < 0.01) and poor (*p* = 0.08) levels, respectively, but the distance swam at IT_100_ and IT_200_ correlated both with RPE (*p* < 0.01 and *p* = 0.04). The [La^−^]peak after IT_100_ showed no correlation with t_Lim_ in IT_100_ (*p* = 0.09), and negative correlation with total distance in IT_100_ (*p* = 0.03). Additionally, the [La^−^]peak after IT_200_ positively correlated with t@90%, t@95%, and O_2Def_ during IT_4x200_ (*p* = 0.03; *p* = 0.05; *p* < 0.01). ⩒O_2_peak and MAV correlated negatively with [La^−^]peak after IT_100_ [*ro* = −0.8, *p* < 0.01, (medium); *ro* = −0.71, *p* = 0.01, (medium)]. MAV correlated negatively with t@95% during IT_200_ (*ro* = −0.59, *p* = 0.04), while ⩒O_2_peak had a positive correlation with total distance in IT_100_ (*ro* = 0.55, *p* = 0.07), as well as ⩒O_2_peak correlating significantly only with Peak-⩒O_2_ during IT_4x200_ [*ro* = 0.64, *p* = 0.02, (medium)] but showed a tendency with Peak-⩒O_2_ during IT_8x100_ (*ro* = 0.52, *p* = 0.09).

**Table 5:** Spearman rank-order coefficients for the responses of temporal, physiological, and perceptual variables between the IT protocols.

Variables	Protocol		RPE		[La^−^]peak	
	IT_100_ vs. IT_200_	IT_8x100_ vs. IT_4x200_	IT_100_	IT_200_	IT_100_	IT_200_
Distance	0.49	*na*	−0.86^**^ [large]	0.60^*^ [medium]	−0.63^*^ [medium]	−0.38
t_Lim_	0.35	*na*	−0.85^**^ [large]	−0.53	−0.52	−0.16
t@VT_2_	−0.04	−0.02	−0.34	0.08	−0.20	0.48
t@90%	0.16	0.23	−0.38	−0.18	0.08	0.64^*^ [medium]
t@95%	0.22	0.30	−0.30	−0.11	0.22	0.59^*^ [medium]
τ	0.62^*^ [medium]	*na*	0.21	0.32	0.32	0.46
O_2_Def	0.59^*^ [medium]	*na*	−0.21	−0.10	0.23	0.72^**^ [medium]
Peak-⩒O_2_	0.20	0.17	0.23	−0.52	−0.29	0.15
[La-]peak	0.74^**^ [medium]	*na*	0.61^*^ [medium]	0.38	*na*	*na*
RPE	0.00	0.67^*^ [medium]	*na*	*na*	*na*	*na*

Obs.: distance (swam at the end of each protocol) in meters; t_Lim_ (time limit); t@VT_2_, t@90%, t@95% (time spent at a given rate) and time constant (τ) all in seconds; O_2Def_ (oxygen deficit at the onset of each IT protocol) in ml/kg; Peak-⩒O_2_ (peak ⩒O_2_ attained during each IT protocol) in ml/kg/min, [La^−^]peak (peak blood lactate accumulation during each IT protocol) in mmol/L; and rate of perceived exertion (RPE; Borg grade of exertion). The acronym “na” refers to the “not analyzed” correlation. Significant correlation at

* *p* ≤ 0.05;

** *p* ≤ 0.01.

## Discussion

This study analyzed the ⩒O_2_ response, as well as the blood lactate concentration and oxygen deficit induced by different intermittent training protocols. The findings support that the two IT formats studied (100-m/15-s and 200-m/30-s performed at MAV) were able to stimulate the exertion level close to maximal ⩒O_2_, as well as moderate to high anaerobic stimulation when considering blood lactate accumulation and oxygen deficit. Therefore, both training protocols showed to elicit physiological responses that were typical of middle-distance swimming performance. Moreover, the analysis of the first 800 m allowed the comparison between both training protocols (IT_8x100_ vs. IT_4x200_), highlighting that the perception of exertion level differed, while performing each IT, with a significantly higher RPE at IT_4x200_.

Other important findings to be highlighted are (i) the Peak-⩒O_2_ attained during both IT protocols did not differ from the ⩒O_2peak_ attained during the incremental step test, which suggests that independently of the IT protocol (*n*x100-m/15-s and *n*x200-m/30-s) a maximal demand upon aerobic contribution was imposed; and (ii) the features of the stimulus upon [La^−^]peak and O_2Def_ denoted a moderate to high reliance on anaerobic contribution, which was similar between the IT protocols and therefore reproduce the energetics required in middle-distance events. In perceptual terms, these IT protocols differed regarding the sensation of exertion, with IT_8x100_ perceived as a less exhaustive exercise, despite no physiological difference between the IT protocols.

To the best of the knowledge of the authors, only two studies analyzed the ⩒O_2_ response in swimming during IT protocols. Libicz et al. (2005) reported that well-trained triathletes spent double the time above 95% of ⩒O_2_max (~145 vs. ~69-s) in 8 × 100-m/30-s than in 16 × 50-m/15-s repetitions, despite the large variability observed in the ⩒O_2_ data constraining the level of statistical significance between each IT. Another study measured time sustained closer to ⩒O_2_max (>90% ⩒O_2_max) during two different IT protocols (16 × 100 vs. 4 × 400 m) performed at submaximal intensity (Δ25%LT-⩒O_2max_; Bentley et al., 2005). Similar to this study, the authors reported no influence of the work interval duration on time sustained above 90% of ⩒O_2_max (~564 vs. 341-s) nor on the maximal ⩒O_2_ (~93 vs. 92%) reached during each IT, as well as reporting no correlation between faster ⩒O_2_K (τ ~17-s during 400-m) and longer times spent closer to ⩒O_2_max. The lack of significance for the differences was attributed to the large variability, which is therefore corroborated to the current data for either total time-limit or time spent at a high ⩒O_2_ in swimming, which was higher than 30% and even larger at higher exercise intensity (90 and 95% ⩒O_2_peak) for both IT.

Notwithstanding, the variability in time-limit performance sounds not to be a matter of sex-related differences in the sample, since other studies including only males or combining male and female participants also reported large temporal variability (Billat et al., 1999; Zuniga et al., 2011), despite sex differences regarding the time limit in intermittent swimming performance remaining to be investigated. For continuous performance in swimming at paces demanding high ⩒O_2_, there are no differences in time limit between male and female swimmers, regardless of conditioning level (Fernandes et al., 2006). Indeed, the exercise tolerance (the endurance performance) during ⩒O_2_ sustained closer to ⩒O_2_peak is determined by the ability of the muscle system to attenuate the reliance on anaerobic sources at the onset of exercise, as well as the accumulation of metabolites, which are processes often analyzed through oxygen deficit, blood lactate accumulation, and ⩒O_2_ on-kinetics (Murgatroyd et al., 2011), with responses to specific exercise but not constrained to sex differences (Billat et al., 1996b; Carter et al., 2006; Reis et al., 2017).

Therefore, the decrease or increase in anaerobic reliance during intermittent exercise relies on the modification of the ratio of work and rest intervals (Billat, 2001; Buchheit and Laursen, 2013b). Interval training has been proposed to increase the time exercising with high ⩒O_2_ demand, which is not attained without a high stimulus on anaerobic glycolysis metabolism (Billat et al., 2000). However, according to Zuniga et al. (2011) and Rønnestad and Hansen (2016), short work intervals (30-s) compared with longer ones (3-min) may allow athletes to complete longer IT exercise sessions with greater metabolic demands and lower [La^−^]. Despite the study of Libicz et al. (2005), which argued that short work interval IT in swimming fails to induce longer time spent near ⩒O_2max_, while inducing an excessive muscular fatigue or acidosis for an effective improvement in endurance and middle-distance performance, this study was the first to evidence this combination.

With regard to the ability of the IT protocols to elicit maximal ⩒O_2_, this study observed that t@VT_2_ (exercise in a severe domain, encompassing time spent at or above VT_2_) is higher than 80% of t_Lim_ to perform either the first 800-m or the entire IT_100_ and IT_200_ protocols, while t@90% comprised 40–50% of time for the first 800-m or t_Lim_ for the entire protocols. Despite that the VT_2_ was attained ~88%⩒O_2_peak in this study, swimming at or above VT_2_ leads to an appreciable increase in VO_2_ (Pessôa Filho, 2012). Hence, the protocols studied enabled the increase in the time spent closer to ⩒O_2_peak, when compared with the findings reported by Sousa et al. (2017) for continuous swimming performance at or above 90%⩒O_2_peak at 90 and 100% of MAV (~78 and ~72%). Moreover, even considering time at or above 90% ⩒O_2_peak for this study in absolute terms (~300–450-s), it was longer than those reported by Sousa et al. (2017; ~268-s). However, in the study of Libicz et al. (2005), the time spent above 95% of maximal ⩒O_2_ was ~22% of total time during IT planned with 8 × 100-m/30-s, which percentage is higher than the ~12–15% of total time observed for t@95% during both IT8x100 and IT4x200 in the present study. It is likely that the mode of performance (continuous vs. intermittent) and rest interval between 100-m bouts (30 vs. 15-s) accounts for the differences between these studies and this study. In spite of the fact that this study only analyzed the effect of velocity at 100% of MAV on ⩒O_2_ demand, the ⩒O_2_ elicited during IT_100_ and IT_200_ has satisfactory high similarity to those reported for continuous or intermittent efforts in swimming (Libicz et al., 2005) and other sports modalities (Billat, 2001; Buchheit and Laursen, 2013b).

Nevertheless, total distance swam and t_Lim_ are not correlated between IT protocols, as well as t@VT_2_, t@90%, and t@95%, which means that the IT protocols are independent in those measures. Also, the protocols did not correlate regarding the peak of ⩒O_2_ reached during the performance of each IT protocol, although temporal ⩒O_2_K and anaerobiosis stimulus (O_2Def_ and [La^−^]peak) correlated with each other between protocols. Therefore, both the protocols are suitable to match middle-distance specificity regarding energetic contribution, which approaches ~25–26 ml·kg^−1^ and ~12 mmol·L^−1^ for swimming (200- and 400-m; Campos et al., 2017). Indeed, the values observed for O_2Def_ and [La^−^]peak in this study are also quite similar to the values reported for IT performed at 100%v⩒O_2max_ in running and cycling (~20–31 ml·kg^−1^; ~5–7 mmol·L^−1^; Billat, 2001; Scott, 2006; Panissa et al., 2018).

However, the IT protocols showed particular correlations with anaerobic variables as follows: (i) negative coefficients between [La^−^]peak vs. ⩒O_2_peak, MAV, t_Lim_, and total distance for IT_100_; and (ii) positive coefficients between [La^−^]peak vs. t@90%, t@95%, and O_2Def_ for IT_200_. These results suggest that swimmers with the highest ⩒O_2_peak and MAV had the tendency to perform IT_100_ with low [La^−^]peak and, hence, tolerate more distance at MAV, which seems to account for the influence of lower perceived exertion reported (less uncomfortable) by those swimmers. In contrast, swimmers with higher MAV had spent less time at or above 90%⩒O_2_peak during IT_200_, suggesting that the improvements of the time at high rates of ⩒O_2_ are related to high ⩒O_2_peak (wide range to ⩒O_2_ adjustments) and anaerobic capacity (enable to support high O_2Def_ and [La^−^]peak), and, therefore, perceived as harder to perform. Hence, the performance during IT_200_ exhibits a typical inverse relationship between MAV and t_Lim_ at rates closer to ⩒O_2_max (Billat et al., 1996a). Additionally, the combination of long exercise bouts (>2 min), high intensity (100% MAV), and short rest intervals (≤30 s), as designed for IT_200_, are difficult to manage with no acidosis because of the reduction in phosphocreatine stores replacement and the increasing reliance on the anaerobic glycolytic contribution (Billat, 2001; Buchheit and Laursen, 2013b).

These dynamics between phosphocreatine nadir and greater glycogen utilization can be more relevant to explain t_Lim_ than microvascular blood flow and muscle oxygen extraction (temporal parameters of ⩒O_2_K). The assumption that t_Lim_ is related to ⩒O_2_K considers that fast VO_2_ response until the targeted muscle O_2_ requirements would reduce O_2_ deficit and metabolite accumulation and, therefore, attenuate phosphocreatine and glycogen stores depletion (Millet et al., 2003b; Bailey et al., 2009). For example, the increase in O_2_ availability induced by prior heavy exercise could be higher for subjects with a slower time constant, improving the time spent above 90%⩒O_2max_ when performing at 100 or 105% of MAV (Millet et al., 2003b). For Bailey et al. (2009), the analysis of ⩒O_2_K has the potential to demonstrate the enhancement of exercise tolerance after interval training through a substantial increase in oxidative energy contribution and a reduced reliance on anaerobiosis stimulus. Despite these authors not finding a correlation between the magnitude of changes in tolerance with time constant of ⩒O_2_K and aerobic conditioning indexes, this could be further explored in future studies trying to gather information on what adjustments in ⩒O_2_K ensure aerobic capacity enhancement, while training with the protocols proposed in this study.

Additionally, the better explanation for the t_Lim_ in IT_200_ is that superior performance was obtained by swimmers with high ⩒O_2_peak, which would delay the attainment of maximal ⩒O_2_ and thus a tendency to reduce the accumulation of metabolites, whereas during IT_100_ the short exercise interval attenuates anaerobic stimulus with no impairment on ⩒O_2_ response. This is in agreement with Zuniga et al. (2011) who reported that short work intervals (30-s) compared with longer ones (3-min) may allow athletes to complete a longer IT session with greater metabolic demands and lower [La^−^] accumulations. The findings of this study might be useful for coaches to decide on the work interval (100- or 200-m bouts) that match the needs for aerobic power of the team. In this study, the inclusion of male and female swimmers is a limitation when considering differences in muscle mass and blood perfusion in the upper limbs (Koons et al., 2019), but how sex differed with regard to ⩒O_2_ increase and tolerance during different work:rest ratio interval training still remains to be answered. Furthermore, swimming with a snorkel and open turns may be a constraint to free swimming training.

## Conclusion

This study concluded that both the IT protocols performed at MAV showed similar physiological and temporal responses whatever the distance (100 or 200-m) utilized for exercise bouts. Additionally, the protocols can be considered suitable to improve middle-distance swimming performance, since both stimulated the exertion level close to maximal ⩒O_2_, as well as moderate to high blood lactate concentrations and oxygen deficit, which is the finding to be highlighted for IT in swimming, as first demonstrated in this study. The fact that only the perceived exertion level differed between the IT protocols suggested that coaches should consider that *n*x100-m/15-s is perceived as less difficult to perform than *n*x200-m/30-s for the first 800-m when managing the best strategy to be implemented for aerobic power enhancement. Finally, the ⩒O_2_K parameters (time constant and amplitude) were not associated to tolerance in each IT protocol, suggesting that t_Lim_ during IT is not related to the parameters of ⩒O_2_K that characterize oxidative contribution and anaerobiosis reliance, but this analysis should be considered to evaluate the potential of aerobic power enhancement with IT.

## Data Availability Statement

The raw data supporting the conclusions of this article are fully available without restriction when required to the authors.

## Ethics Statement

This study was approved by the local University Ethical Committee of the Faculdade de Motricidade Humana (UL-CEFMH: 39/2015). Written informed consent to participate in this study was provided by the participants’ legal guardian/next of kin.

## Author Contributions

TA, DP, and FA conceived and designed the study. TA, DP, ME, JR, AS, DM, and FS conducted the experiments and analyzed the data. TA, DP, ME, JR, and FA wrote the manuscript. All authors contributed to the article and approved the submitted version.

### Conflict of Interest

The authors declare that the research was conducted in the absence of any commercial or financial relationships that could be construed as a potential conflict of interest.

## Supplementary Material

Supplementary Material Transportation of gas analyzer system and sampling unit at the side of the pool.
